# A case report of pulmonary artery sling and situs inversus incompletes

**DOI:** 10.1097/MD.0000000000024021

**Published:** 2021-01-22

**Authors:** Yingchun Xu, Dan Xu, Beilei Cheng, Lanfang Tang, Zhimin Chen, Lizhong Du

**Affiliations:** a Department of Pulmonology; b Department of Neonatology, The Children's Hospital, Zhejiang University School of Medicine, National Clinical Research Center for Child Health, Hangzhou, Zhejiang Province, China.

**Keywords:** pulmonary artery sling, situs inversus incompletus, airway anomalies, tracheoplasty

## Abstract

**Rationale:**

Pulmonary artery sling (PAS) is a rare congenital anomaly. Associated airway anomalies and/or those of the cardiovascular system are present in about half the patients. Situs inversus is a rare disease in which organs of the chest and/or abdomen are arranged in a mirror image reversal of their normal position. Herein, we report a rare case of pulmonary artery sling and situs inversus incompletus, which has not yet been reported.

**Patient concerns:**

A 10-year-old girl was admitted because of heart murmur for more than 9 years. On physical examination, the second heart sound was prominent, and a grade 2/6 systolic murmur was heard at the left mid-sternal border. Echocardiography revealed PAS and atrial septal defect (8.6 mm). A chest computer tomography angiograph demonstrated that she had lung inversus, right aortic arch, and right lung hypoplasia in addition to PAS, with a normal positioning of the heart. The PAS intersected and twisted across the bronchus, which was obviously narrowed. The PAS was type II B, since the carina was at the T6 level without a separate right upper lobe bronchus.

**Diagnoses:**

Her final diagnosis was that of PAS, tracheal stenosis, situs inversus incompletus, right lung hypoplasia, right aortic arch, ASD and PDA.

**Interventions:**

She underwent one-stage total correction for her initial cardiovascular defects through median sternotomy under cardiopulmonary bypass support.

**Outcomes:**

She had an uneventful recovery and completely healthy following the procedure.

**Lessons:**

A thorough examination before PAS surgery was essential in discovering and carefully evaluating complicated heart and lung anomalies.

## 1 Introduction

Pulmonary artery sling (PAS) is a rare congenital anomaly in which the left pulmonary artery (LPA) originates from the posterior aspect of the right pulmonary artery (RPA) and courses over the right main bronchus and then posteriorly crosses between the trachea and esophagus to reach the left lung, thereby forming a partial sling around the trachea.^[1]^

The normal arrangement of asymmetric organs (heart an great vessels, lungs, liver, gallbladder and biliary tract, spleen) is termed “situs viscerum solitus”, whereas the mirror-image of this arrangement is called situs inversus.^[2]^ Situs inversus is a rare disease. Total situs inversus is usually asymptomatic. If patients have some organs in their usual position and some reversed, it is known as situs inversus incompletus or partial situs inversus.^[2]^ The incidence of situs inversus incompletus is less than 1 in 22,000.^[3]^

Herein, we report on a case of PAS and situs inversus incompletus, with a series of cardiovascular abnormalities, which have not been reported yet. The study was approved by the Children's Hospital of Zhejiang University School of Medicine. The patient and parents have provided informed consent for publication of the case.

## 2 Case presentation

**Figure 1:**
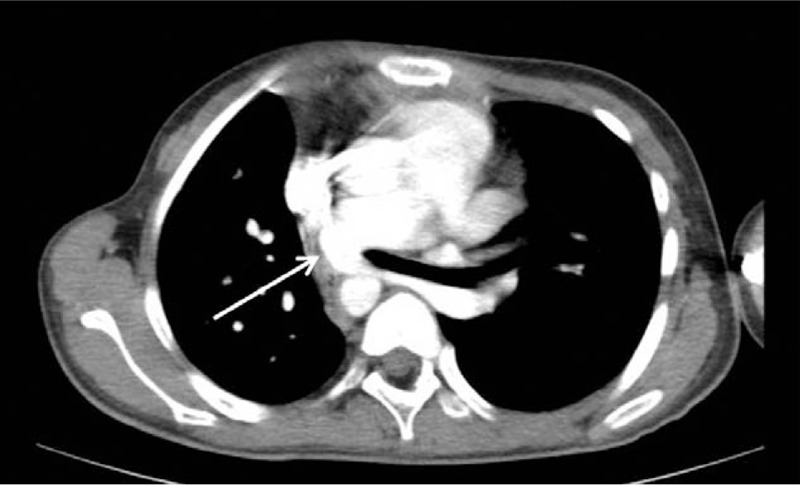
The chest computer tomography angiographic shows that the left pulmonary artery originates from the right pulmonary artery and goes around behind the trachea to the left (white arrow). The mediastinum is slightly pushed to the right of the chest.

**Figure 2:**
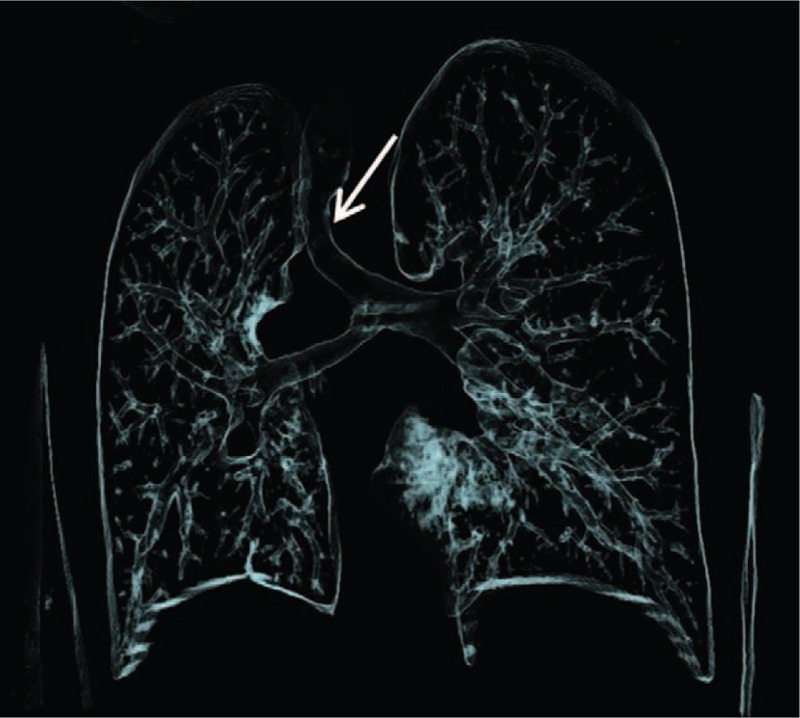
Reconstructed image shows that the trachea is twisted and narrow (white arrow). The left lung has 3 while the right lung has 2 lobes. The right lung is small.

A 10-year-old girl was admitted on July 21, 2014 because of a history of heart murmur for more than 9 years in July, 2014. She had no significant symptoms, and absence of cough, wheezing, or dysphagia. Patient was diagnosed with PAS by echocardiography soon after she was born. The parents refused elective surgery due to poverty at that time. She had recurrent respiratory infections before 5 years old and “healthy” afterward. The patient had no personal or family history. Physical examination upon admission revealed that the second heart sound was prominent, and a grade 2/6 systolic murmur was heard at the left mid-sternal border during physical examination. A recent echocardiography revealed PAS and atrial septal defect (ASD) (8.6 mm). Laboratory examinations showed that complete blood count, prothrombin and partial thromboplastin times, D-dimers, serum C-reactive protein, blood biochemistries, arterial blood gas, and urinalysis were all normal. After she was admitted, a chest computer tomography angiographic (CTA) demonstrated that she had lung inversus, right aortic arch and right lung hypoplasia in addition to PAS, with a normal positioning of the heart (Fig. 1). The PAS crossed and twisted the bronchus, which was obviously narrowed (Fig. 2). It was determined to be a type II B PAS since the carina was at the T6 level without a separate right upper lobe bronchus.^[1]^ The trachea was narrow in the lower part. The electrocardiogram and abdomen ultrasound were normal.

The patient underwent a one-stage total correction of her initial cardiovascular defects through median sternotomy under cardiopulmonary bypass support. During the surgery, a patent ductus arteriosus (PDA) (3.0 mm) was also found. The left pulmonary artery (LPA) was dissected, and reimplanted to the main pulmonary artery at a normal take-off site of the LPA. ASD and PDA were amended at the same time.

The final diagnosis was PAS, tracheal stenosis, situs inversus incompletus, right lung hypoplasia, right aortic arch, ASD, and PDA. Patient had an uneventful recovery after surgery and was discharged without respiratory difficulty on breathing room air. She was subsequently followed-up for 5 years. She remained completely healthy following discharge, without evidence of pneumonia, recurrent respiratory infections or eating difficulties. In addition, the echocardiogram was normal.

## 3 Discussion

The anatomical arrangement of human organs might be described using the following categories: situs solitus; situs inversus; and situs ambiguous. The normal position of the thoracic and abdominal viscera is referred to as situs solitus. Situs inversus is one of the abnormalities with an incidence of 1:5000 to 1:20,000.^[4]^ Situs inversus is classified as either situs inversus totalis or situs inversus incompletus.^[5]^ In situs inversus, if the heart is switched to the right side of the thorax, it is known as “situs inversus with dextrocardia” or “situs inversus totalis”. If the heart remains on the normal left side of the thorax, a much rarer condition is apparent, which is known as “situs inversus with levocardia” or “situs inversus incompletus”. In this case, the lung anatomy is reversed in that the left lung has 3 lobes while the right lung has 2 lobes. The heart remains on the left side of the thorax. Thus, the patient described in this report presented with situs inversus incompletus. The pathogenesis of situs inversus is not yet clear. In situs inversus incompletus, there may be only thoracic inversion, cardiac chamber reversal, or only abdominal organ inversion accompanied by syndromes showing splenic anomalies, an annular pancreas, a horseshoe kidney, a diaphragmatic hernia, or other developmental abnormalities. Situs inversus may due to a possible abnormality in rotation in early embryonic life.^[6]^ Situs inversus totalis is quite compatible with life while situs inversus incompletus often accompanies with cardiopulmonary anomalies.

PAS is a rare congenital malformation. Associated anomalies of the tracheobronchial tree and/or of the cardiovascular system are present in about 50% of patients.^[7]^ Further, approximately two-thirds of PAS cases are associated with intrinsic tracheal stenosis with complete cartilaginous rings.^[1]^ In this case, a series of anomalies were combined together, which has not been reported elsewhere, at least to the best of our professional knowledge. Cardiovascular anomalies were PAS, right aortic arch, ASD, and PDA, which were all witnessed during surgery. Respiratory anomalies were tracheal stenosis, situs inversus incompletus, and right lung hypoplasia. However, it was fortunate that none of the observed manifestations seen during surgery affected her cardiopulmonary function. The heart murmur was due to ASD. Besides this, she was almost healthy, without any evidence of recurrent respiratory pneumonia, feeding difficulties, weakness or activity limitation. She was completely healthy postsurgery. Compared to doing surgery immediately following birth, we think that it might be suitable for her to have waited until achieving teenage years.

Situs inversus can pose a challenge during surgery because of the extraordinary anatomy of these patients. Some surgeons have recommended that the operator and assistant positions should be reversed, especially during laparoscopic surgeries.^[8]^ In endoscopic retrograde cholangiopancreatography procedures, 2 methods were suggested in patients with situs inversus: the endoscope is rotated 180° clockwise in stomach and another 180° clockwise in duodenum; or the patients placed in the right prone position and the endoscopist performs the procedure form the left side of the table.^[9]^ In this case, the patient has cardiopulmonary deformities without abnormalities in the abdomen. She underwent an open chest surgery through median sternotomy. Situs inversus has little effect on doing the surgery. Because the patients has little symptoms, narrowed bronchus was left untreated. She remain asymptomatic after the surgery.

Herein a patient with pulmonary artery sling and situs inversus incompletus was reported. A thorough examination before surgery was essential to discovering and carefully evaluating complicated heart and lung anomalies. For patients with complicated cardiopulmonary anomalies but with minimal presenting symptomology, an appropriate time for elective surgery should be discussed.

## Author contributions

**Conceptualization:** Lizhong Du.

**Data curation:** Yingchun Xu, Dan Xu, Beilei Cheng.

**Formal analysis:** Beilei Cheng, Lanfang Tang.

**Investigation:** Yingchun Xu, Dan Xu.

**Methodology:** Zhimin Chen, Lizhong Du.

**Project administration:** Zhimin Chen, Lizhong Du.

**Writing – original draft:** Yingchun Xu, Dan Xu, Beilei Cheng.

**Writing – review & editing:** Lizhong Du.
